# Coordination Environment of Cu(II) Ions Bound to N-Terminal Peptide Fragments of Angiogenin Protein

**DOI:** 10.3390/ijms17081240

**Published:** 2016-08-01

**Authors:** Antonio Magrì, Alessia Munzone, Massimiliano Peana, Serenella Medici, Maria Antonietta Zoroddu, Orjan Hansson, Cristina Satriano, Enrico Rizzarelli, Diego La Mendola

**Affiliations:** 1 Institute of Biostructures and Bioimages, National Council of Research ( CNR), Via P. Gaifami 18, 95126 Catania, Italy; leotony@unict.it (A.M.); erizzarelli@unict.it (E.R.); 2 Department of Chemical Sciences, University of Catania, Viale A. Doria 6, 95125 Catania, Italy; alemunzy31@hotmail.it; 3 Department of Chemistry and Pharmacy, University of Sassari, Via Vienna 2, 07100 Sassari, Italy; peana@uniss.it (M.P.); sere@uniss.it (S.M.); zoroddu@uniss.it (M.A.Z.); 4 Department of Chemistry and Molecular Biology, University of Gothenburg, Medicinaregatan 9C, 41390 Göteborg, Sweden; orjan.hansson@chem.gu.se; 5 Department of Pharmacy, University of Pisa, Via Bonanno Pisano 6, 56126 Pisa, Italy

**Keywords:** copper, angiogenesis, recombinant protein, peptidomimetic, confocal microscopy, actin, stability constants, NMR, neuroblastoma cells

## Abstract

Angiogenin (Ang) is a potent angiogenic factor, strongly overexpressed in patients affected by different types of cancers. The specific Ang cellular receptors have not been identified, but it is known that Ang–actin interaction induces changes both in the cell cytoskeleton and in the extracellular matrix. Most in vitro studies use the recombinant form (r-Ang) instead of the form that is normally present in vivo (“wild-type”, wt-Ang). The first residue of r-Ang is a methionine, with a free amino group, whereas wt-Ang has a glutamic acid, whose amino group spontaneously cyclizes in the pyro-glutamate form. The Ang biological activity is influenced by copper ions. To elucidate the role of such a free amino group on the protein–copper binding, we scrutinized the copper(II) complexes with the peptide fragments Ang(1–17) and AcAng(1–17), which encompass the sequence 1–17 of angiogenin (QDNSRYTHFLTQHYDAK-NH_2_), with free amino and acetylated N-terminus, respectively. Potentiometric, ultraviolet-visible (UV-vis), nuclear magnetic resonance (NMR) and circular dichroism (CD) studies demonstrate that the two peptides show a different metal coordination environment. Confocal microscopy imaging of neuroblastoma cells with the actin staining supports the spectroscopic results, with the finding of different responses in the cytoskeleton organization upon the interaction, in the presence or not of copper ions, with the free amino and the acetylated N-terminus peptides.

## 1. Introduction

Human Angiogenin (Ang) is a 14 kDa protein belonging to the ribonucleases family, with a RNase catalytic activity about 10^6^ lower than pancreatic RNase [1].

Ang is a physiological constituent of human plasma (in a concentration range of 250–360 µg/L) but is over-expressed in patients affected by different types of cancers. Indeed, Ang has been discovered and isolated for the first time from a colon adenocarcinoma cell line [2,3]. The protein influences nearly all steps of tumorigenesis, including cell proliferation, migration and metastatic progression [3,4,5].

Ang is among the most potent angiogenic factors known thus far, as determined in various models, both in vitro and in vivo [6,7]. Moreover, Ang regulates the expression of other angiogenic factors, such as the vascular endothelial growth factor (VEGF), the epidermal growth factor (EGF), the acidic fibroblast growth factor (aFGF) and the basic fibroblast growth factor (bFGF) [8,9,10,11,12].

The angiogenic activity of this protein occurs through a series of events, involving: (i) the Ang ribonucleolytic activity; (ii) the stimulation of basement membrane degradation; (iii) the stimulation of signal transduction; and (iv) the nuclear translocation [13,14]. From a molecular point of view, three distinct regions of the protein are necessary: (a) the catalytic site for RNase activity that involve the residues His-13, Lys-40 and His-114; (b) the putative cell binding region, encompassing the residues 60–68 (KNGNPHREN sequence); and (c) the nuclear translocation residues 31–35 (RRRGL sequence) [5,13,14].

The angiogenin functions are also exerted extracellularly, where the protein activates signal-related kinase1/2 (ERK1/2) in human umbilical vein endothelial cells (HUVECs) or stress-associated protein kinase/c-Jun N-terminal kinase (SAPK/JNK) in human umbilical artery smooth muscle cells (HuASMCs) [15,16]. The binding of Ang to a putative 170 kDa receptor has been found in HUVEC [17], but a possible receptor role has been suggested also for other proteins, including actinin [18], follistatin [19] fibulin 1 [20] and actin [21,22,23].

In particular, the formation of a high-affinity complex between Ang and actin has been reported, although the actin-binding site on Ang is still unknown [22,23]. The interaction of actin with Ang induces changes in the cell cytoskeleton, remodeling of the extracellular matrix (ECM) and the degradation of basement membrane, thus promoting cell invasion into the perivascular tissue [22,23]. A possible activity of Ang towards actin aggregation has been indicated and related to the tendency of the protein to form multivalent intermolecular interactions. Interestingly, the Ang residues involved in such processes are localized in the N- and/or C-terminal domains of the protein [22].

It has to be noted that Ang’s functional role is not limited to the angiogenesis stimulation, since the protein is widely expressed in all mammalian organs and tissues [5,12]. For instance, the protein exists at high concentrations in motoneurons, where it is involved in the onset of amyotrophic lateral sclerosis (ALS) [24,25]. In addition, Ang is down-regulated in patients affected by Alzheimer’s diseases [26] and in α-synuclein mouse model of Parkinson’s disease [27].

Copper is also an angiogenic factor in vivo [28,29,30] and plays a role in the progression of different cancers as wells as in the onset/progression of neurodegenerative diseases [31,32,33,34]. Moreover, during angiogenesis process, copper translocates from intracellular to extracellular space [35]. In this pathway, still unknown, it is very likely that copper acts as signaling factor, by means of the binding with extracellular proteins involved in angiogenesis, such as Ang. Furthermore, the binding affinity between human angiogenin and endothelial cells is largely increased in the presence of copper ions [36,37]. Different peptide fragments encompassing either the putative binding site or the N-terminal domain of angiogenin have been demonstrated to be able to bind copper ions tightly [38,39].

Previous studies relied on the hypothesis that the angiogenic activity of copper and Ang occurred through different and independent biological pathways [40]. Recently, a strong correlation between the protein and the metal ion has been demonstrated: Cu^2+^ increases the expression of Ang and modulates its intracellular localization in HUVEC, affecting angiogenic activity of protein and ERK activation signaling [41].

This contrast is likely related to the use of different forms of the Ang protein: the recombinant (r-Ang), mostly used in many literature reports, containing an extra methionine as first residue, and the native form, present in vivo (“wild-type”-angiogenin, wt-Ang), which lacks the free amino terminal group, owing to the spontaneous cyclization of the first glutamine group residue to pyroglutamate [1]. 

Noteworthy, the two proteins, r-Ang and wt-Ang, bind copper ions differently: in the recombinant form, the anchoring site of metal ion is the terminal amino group, whereas the native wild-type protein binds Cu^2+^ through His-114 and His-13 [42]. Such amino acids constitute two out of the three catalytic residues of the protein, and the addition of copper ions at physiological pH influences much more the RNase activity and the capillary-like tubes formation in wt-Ang than in r-Ang [42]. A comprehensive characterization of the Ang–copper(II) complex species is therefore a valuable support in the improved understanding of potential mutual biological influences. 

In this work, we report on the copper(II) complexes formed with the peptide fragment encompassing the sequence 1–17 (QDNSRYTHFLTQHYDAK-NH_2_; named Ang(1–17)) and its acetylated form (Ac-QDNSRYTHFLTQHYDAK-NH_2_; named AcAng(1–17)). A comprehensive chemical characterization by means of potentiometry, nuclear magnetic resonance (NMR), ultraviolet visible (UV-vis) and circular dichroism (CD) spectroscopies is presented to depict the copper coordination environment in the N-terminal domain of the protein. The obtained data demonstrate the role of N-terminal free amino group in the metal binding. Moreover, the effect of Ang(1–17) and AcAng(1–17) peptides on the actin aggregation has been tested on human neuroblastoma cells, in the presence or not of copper ions, to exploit their potential use as mimicking system of whole r-Ang and wt-Ang proteins, respectively.

## 2. Results

### 2.1. Conformational Features of Ang(1–17) and AcAng(1–17) Peptides

The five and seven protonation constant values, respectively, of Ang(1–17) and AcAng(1–17) peptides, as determined by potentiometric titrations, are reported in Table 1.

In the investigated pH range, Ang(1–17) and AcAng(1–17) have a total of eight and seven protonation centres, respectively. This difference is related to the amino group in the N-terminal amino acid residue, free in Ang(1–17) and blocked by acetylation in AcAng(1–17). However, due to precipitation phenomena observed at pH = 9 during the titrations of Ang(1–17), the three protonation constant values of Tyr and Lys side chains for this ligand were not determined.

For both peptides, the two lowest p*K*s refer to the carboxylic group of the two aspartic residues. Since their deprotonation occurred with overlapping titration curves, the first and second protonation constants have not been assigned to the specific residue, Asp-2 or Asp-15, respectively [43]. The next two deprotonation steps involve the imidazole nitrogen atoms of the two His residues; also in this case hence each protonation constant has been considered as a macroconstant value, due to the partially overlap of the titration curves,. Until the deprotonation reaction step of histidine (pH < 7), the behavior of the two peptides is similar. At higher pH values, the deprotonation processes of Tyr-6, Tyr-14 and Lys-17, measured only for AcAng(1–17), resulted in the assignment of three macroconstant values, in good agreement with those observed for analogous peptide fragments [39,44].

**Figure 1:**
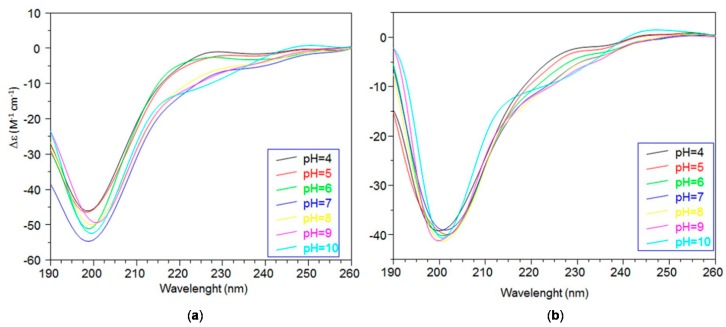
Far ultraviolet-circular dichroism (Far-UV CD) spectra of: (**a**) Ang(1–17); and (**b**) AcAng(1–17).

The far-UV CD spectra of Ang(1–17) and AcAng(1–17) obtained in the pH range of 4–10 are shown in Figure 1.

At acidic pH, for both peptides, the presence of a band with a minimum at 198 nm and a maximum at 228 nm suggest a prevalent random coil conformation. 

Ang(1–17) displays a slight increase of the signal at 198 nm and a decrease of that at 228 nm at increasing pH values. Above pH 7, the histidine residues are deprotonated and the spectra show a decrease of the minimum and the appearance of two new bands displaying a maximum at 232 nm and a minimum at 242 nm. These effects are more evident at pH 10 where the 198 nm band is shifted at 201 nm and a new broad band is observed at 248 nm, assigned to the deprotonated phenolate group of tyrosine residues [44]. All these features point to conformational changes of Ang(1–17), likely due to the electrostatic charges, and regulated by the pH change through different protonation states of the peptide.

As to AcAng(1–17), the curve changes are less prominent in comparison to Ang(1–17).

The ligands have been also characterized by means of one dimensional (1D) ^1^H, two dimensional (2D) ^1^H-^13^C Heteronuclear Single-Quantum Correlation (HSQC), 2D ^1^H-^1^H Total Correlation Spectroscopy (TOCSY) and 2D ^1^H-^1^H Rotating-frame Overhauser Spectroscopy (ROESY) NMR measurements at pH values of 5.5 and 7. All signals have been assigned (Tables S1–S8 in Supplementary Materials); the range of ppm chemical shift related to peptide backbone N–H protons (7.79 < HN < 8.42) is narrower for both Ang(1–17) and AcAng(1–17) in comparison to that reported for the whole protein (7.74 < HN < 8.62) [42]. However, such a shift is larger than that assigned to completely unfolded protein (8.12 < HN < 8.42) [45], suggesting a partial folding of both peptides. In Figure S1, the comparison of the spread HN chemical shift (ppm) values with respect to a random coil conformation are reported for the fragment peptides Ang(1–17) and AcAng(1–17), respectively.

### 2.2. Far ultraviolet-Circular Dichroism (Far-UV CD) Study of Copper Complexes with Ang(1–17) and AcAng(1–17) Peptides

**Figure 2:**
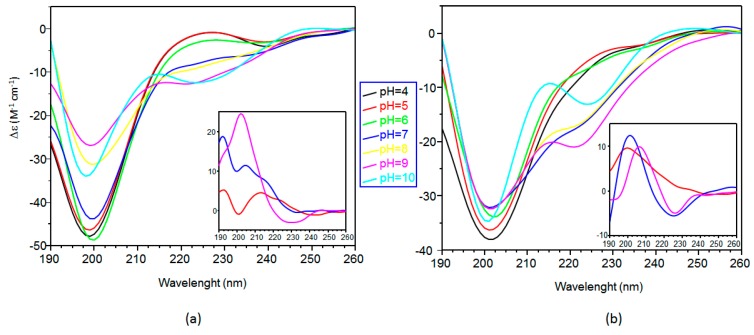
Far-UV CD spectra of copper(II) complexes with ligands (L): (**a**) Ang(1–17); and (**b**) AcAng(1–17), [L] = 5 × 10^−5^ M; metal to ligand molar ratio of 1:1. Insets: the spectra of copper complexes subtracted by the spectra of the free ligands.

The secondary structure of the peptides, correlated to the far-UV CD spectra features, visibly changes upon the addition of copper ions (Figure 2). Specifically, for Ang(1–17), one equivalent of copper(II) induces a decrease for the signal at 198 nm in the pH ranges of 4–5 and 7–9, whereas a opposite trend is observed at pH 6 and 10, accompanied also by a red-shift at pH = 6 and a blue-shift at pH = 10, respectively (Figure 2a). Moreover, at the highest pH values of 9 and 10, two new maxima and one minimum are clearly visible, at 214, 245 and 224 nm, respectively.

The CD difference spectra (inset in Figure 2a) do not indicate an increase of specific conformational structure at the different pH values, but the presence of a turn structure can be figured out at the highest pH values of 9 and 10, as reported for other linear peptides [46].

The enhancement of the peptide turn conformation, induced by the pH increase, can be interpreted as due to the involvement of backbone amide nitrogen atoms in Cu^2+^ coordination (see Section 2.3). The far-UV CD spectra of Cu-peptide system are governed by the amide chromophore, but may contain a contribution of aromatic side chains, in particular the band at 224 and 245 nm might be related to the tyrosine phenolic group deprotonation [47].

On the other hand, by addition of one equivalent of copper to AcAng(1–17), a general decrease and slight shifts of the bands at 198 and 228 nm in the whole pH range of 4–9 are observed (Figure 2b). The difference spectra indicate, already at acidic pH, the formation of turn structures that increase at basic pH. The intensity of signals is much lower than that observed for Cu^2+^–Ang(1–17). Indeed, in the acetylated peptide, the imidazole nitrogen atoms of His-8 and His-13 are the potential metal anchoring sites; hence, the formation of a macrochelate, resulting in a turn structure, might include primarily the central portion of the peptide (residues 8–13). Otherwise, in Ang(1–17), the free amino terminal amino group is a further potential copper anchoring site; the stronger dichroic effect observed suggests the formation of a greater macrochelate involving amino group of first residue and imidazole nitrogen of His-8 and/or His-13.

### 2.3. Speciation and Characterization of Copper Complexes with Ang(1–17) and AcAng(1–17) Peptides

**Figure 3:**
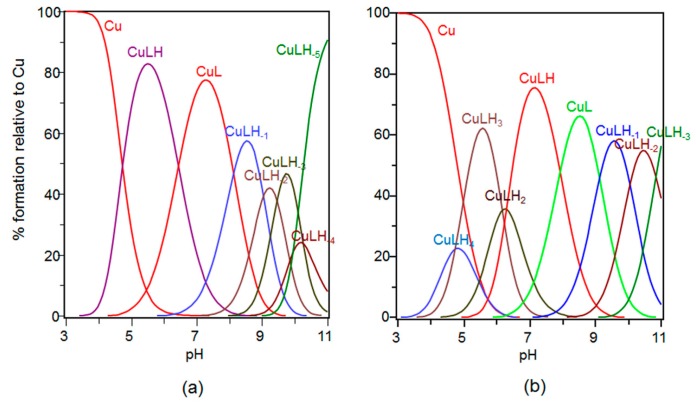
Species distribution of copper(II) complexes with: (**a**) Ang(1–17); and (**b**) AcAng(1–17). [L] = 1 × 10^−3^ M; metal to ligand molar ratio of 1:1.

The stability constant values are reported in Table 2 and the corresponding distribution diagrams in Figure 3, respectively.

The distribution diagram in Figure 3a shows that [CuLH] is the first copper(II) complex species formed by Ang(1–17). The logK value determined for this species (log*K* = logβ_111_ − logβ_011_ = 6.05) suggests the involvement of two nitrogen atoms and a 2N_2_O coordination mode, in good agreement with data reported for analogous peptide sequences [48]. Different isomers involved as copper(II) anchoring sites, either the N-terminal amino group and one imidazole nitrogen or two imidazole nitrogens are likely. 

UV-vis and CD parameters can discriminate the actual copper(II) coordination environment (Table 3). The UV-vis parameters of [CuLH] species (λ_max_ = 628 nm ε = 90 M^−1^·cm^−1^, see Table 3) rule out the formation of a macrochelate, involving two imidazole nitrogen atoms and one carboxylate, which would exhibit the absorption at higher wavelength [49]. Our data are indeed very similar to those reported for a peptide binding Cu^2+^ by means of the terminal amino group, the deprotonated amide nitrogen atom and the oxygen of a carboxylate group of contiguous aspartic residue [50].

Accordingly, the CD spectrum carried out at pH = 5.5 shows the presence of a band at 299 nm ascribable to a N-amide →Cu^2+^ charge transfer, confirming the presence of a deprotonated amide nitrogen in metal coordination environment. In addition, a band at 265 nm is found, assigned to either imidazole or amino groups. Hence, the [CuLH] species has to be considered instead as a [CuLH_−1_(H)_2_] species, in which one imidazole nitrogen is still protonated and the actual chromophore is Cu(NH_2_, N^−^, O_COO−_, O_water_).

The next species formed, [CuL], is predominant at physiological pH. The stepwise constant log*K*_110_ (logK_110_ = logβ_110_ − logβ_111_ = 6.35) is compatible with a deprotonation of another amide nitrogen atom. The 25 nm blue shift in the UV-vis λ_max_ absorption confirms the involvement of a further nitrogen atom in the metal coordination environment.

In Cu^2+^ 3N1O coordination mode two or more isomers may exist; among them the most likely are the following: (i)-form where a second deprotonated amide atom is coordinated to Cu^2+^ (NH_2_, 2N^−^, O_COO−_); and (ii)-form, with a deprotonated imidazole ring (N_im_) in the equatorial plane (NH_2_, N^−^, N_Im_, O_COO−_). The presence in the CD spectra recorded at pH = 7 of a dichroic band centered at 336 nm (diagnostic of N-imidazole →Cu^2+^ charge transfer) point to the predominant (ii)-form. Indeed, the UV-vis parameters (λ_max_ = 605 nm ε = 95 M^−1^·cm^−1^) confirm this coordination mode, as found in literature for complex species formed by peptides with a similar primary sequence [49]. 

Increasing the pH, [CuLH_−1_] complex species is formed; this species displays its maximum percentage of formation at pH = 8.5. The log*K* value (log*K* = logβ_11-1_ − logβ_110_ = 8.14) indicates deprotonation and a further coordination of an amide nitrogen atom. This hypothesis is confirmed by UV-vis spectrum where a 35 nm blue shift of the d-d band is observed (Table 3), and by the intensity increase of the dichroic band relative to N-amide →Cu^2+^ charge transfer, at 322 nm.

The next deprotonation species formed is [CuLH_−2_], at pH ~9, closely followed, at pH ~9.5, by the formation of [CuLH_−3_]. The UV-vis spectra recorded at in the pH range 9–10 result as the superposition of three complex species, namely [CuLH_−1_], [CuLH_−3_], and [CuLH_−3_]. However, based on the potentiometric data, the deprotonation of a third amide nitrogen atom in the [CuLH_−2_] species can be assumed, with the formation of a complex where the metal ion is bound to four nitrogen atoms [48,49,50]. As side comment, it is interesting to note that Cu(II) coordination to Ang(1–17) prevented the precipitation of peptide at basic pH values, as instead observed for its apo-form.

Above pH = 10, the Tyr and Lys deprotonation occurs, with the formation of [CuLH_−3_], [CuLH_−4_] and [CuLH_−5_] species. Spectroscopic data do not evidence any change, indicating that Tyr and Lys residue are not involved in metal ion binding [48].

As to AcAng(1–17), Figure 3b shows that the peptide forms the [CuLH_4_] species at pH = 4, which reaches its maximum percentage of formation (around 25%) at pH = 5. The calculated stability constant (log*K* = logβ_114_ − logβ_014_ = 3.99) is higher respect to the typical stability constant of a copper complex in which the metal ion is coordinated to only one imidazole nitrogen of a histidine [51]. Accordingly, this complex species involves a carboxylate group of one aspartate residue, whereas the other aspartate is deprotonated but not coordinated to the metal. Therefore, four isomers with [CuLH_4_] stoichiometry can exist, formed by His-8, His13, Asp-2 and Asp-15. The smaller macrochelate formed by His13 and Asp-15 is the most thermodynamically favored isomer form. 

Increasing the pH, [CuLH_3_] is formed and its stability constant (log*K* = logβ_131_ − logβ_031_ = 5.95) is indicative of a macrochelate formation with coordination to Cu^2+^ of two imidazole rings and one COO^−^ group of aspartic. This stability constant value is in a good agreement with the value reported for the corresponding complex species formed by other similar peptide fragments [39]. 

The UV-vis parameters of [CuLH_3_] species (λ_max_ = 650 nm, ε = 60 M^−1^·cm^−1^, see Table 3) indicate a metal coordination to two imidazole nitrogen atoms, one carboxylate group and a water molecule. It is to note that for the corresponding species formed by the unacetylated peptide, λ_max_ value is at the lower wavelength of 628 nm. The CD spectra at pH 5.9 do not show any evidence of the amide band while a band at 331 nm is a fingerprint of imidazole coordination.

The next deprotonation step involves one amide backbone nitrogen atom, but the [CuLH_2_] is only a minor species; for this reason, no spectroscopic data for this complex species have been obtained.

At physiological pH, the main species for the Cu-AcAng(1–17) system is [CuLH]. The calculated p*K* value (p*K*(2/1) = 5.79; see Table 2) indicates the deprotonation of a further amide nitrogen. This finding suggests the simultaneous presence of the imidazole rings and amide nitrogen atoms bound to the copper(II), as confirmed by the CD spectrum recorded at pH = 7.4, showing the presence of two bands centered around 360 nm and 320 nm, respectively. Furthermore, UV-vis parameters (λ = 580 nm and ε = 10^5^ M^−1^·cm^−1^) indicate the involvement of four nitrogen atoms in the equatorial plane of the metal.

Increasing the pH, the [CuL] species is formed. In this case, the p*K* determined for this deprotonation step is 7.38 and is reported in Table 2 as p*K*(1/0). The coordination of a third nitrogen atom deprotonated is confirmed by the blue-shift of 40 nm observed for the λ_max_ (λ = 540 nm and ε = 125 M^−1^·cm^−1^). Furthermore, the increase of the ε is a clear indication that the coordination to the Cu(II) of this third amide nitrogen atom deprotonated with a release of an imidazole ring, created a more distorted copper coordination polyhedron with a stronger ligand field.

The other complex species formed at basic pH is assigned to the deprotonation of the side chains of the tyrosine and lysine residues that do not influence the complexation of Cu(II), as shown by the unchanged CD ad UV-vis spectral parameters.

### 2.4. Nuclear Magnetic Resonance (NMR) Study of Peptide-Copper Complexes

Peptides were scrutinised by NMR to determine the specific anchoring site and to discriminate among the different possible copper complexes isomers formed by Ang(1–17) and AcAng(1–17). Selective paramagnetic line broadening and signal disappearance in the 1D ^1^H, 2D ^1^H-^13^C HSQC and 2D ^1^H-^1^H TOCSY NMR spectra were recorded at pH 5.5 and 7, and at different copper to ligand molar ratios. Depending on the distance of the paramagnetic center, the observation of proton signals of the residues close to the metal site may be precluded, due to the broadening of NMR signals [52]. Only sub-stoichiometric amounts of Cu^2+^ ion solution were added to both the peptide solutions, in order to follow the relaxation effect of any nucleus that is in closer vicinity to the paramagnetic center. The line-broadening effects were taken into account in order to localize the metal binding sites along the sequence of the peptides.

At pH 5.5, by addition of Cu(II), a gradual but specific decrease and disappearance of proton signal intensity from H8, H13 and D15 residues and, to a lesser extent, of protons from F9 and Y14 residues, are detected in the ^1^H NMR spectra of AcAng(1–17). The superposition of ^1^H aromatic and aliphatic region for AcAng(1–17) is reported in Figure S2.

**Figure 4:**
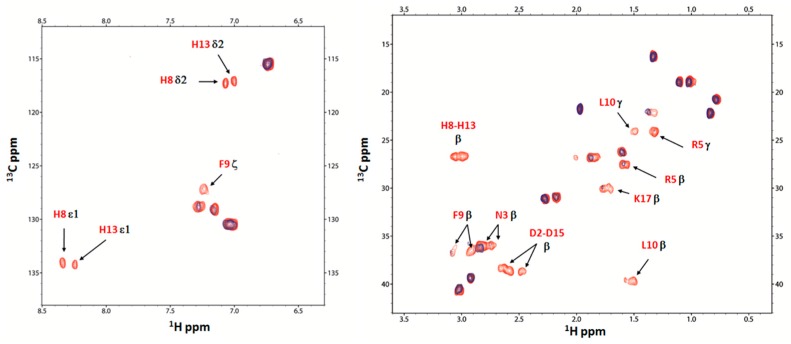
Aromatic and aliphatic region of ^1^H-^13^C HSQC spectrum of AcAng(1–17) free (red) and AcAng(1–17):Cu(II), 1:0.02 mole ratio (blue), at pH 5.5. Disappearing peaks and those with major broadening have been labelled.

Figure 4 shows the comparison of 2D ^1^H-^13^C HSQC NMR spectra, in the aromatic and aliphatic region, of AcAng(1–17) free (red) with Cu(II):AcAng(1–17) complexes at 0.02/1 molar ratio (blue) at pH 5.5.

The disappearance of signals from Hε1 and Hδ2 of H8 and H13 residues together with Asp-15 residue is visible, supporting the concomitant participation of both histidine residues together with aspartate residue in the coordination to Cu^2+^ ions. Moreover, the attenuation of signals intensities owing to residues close to the paramagnetic binding site is detected for the residues F9, L10, Q12 and Y14.

The direct involvment of D15 residue in the Cu^2+^ coordination, rather than D2, is also supported by the disappearance in the ^1^H-^1^H TOCSY spectrum of its Hβ-HN and Hα-HN correlations (Figure S3), confirming the representation of [CuLH_3_] coordination mode above described.

**Figure 5:**
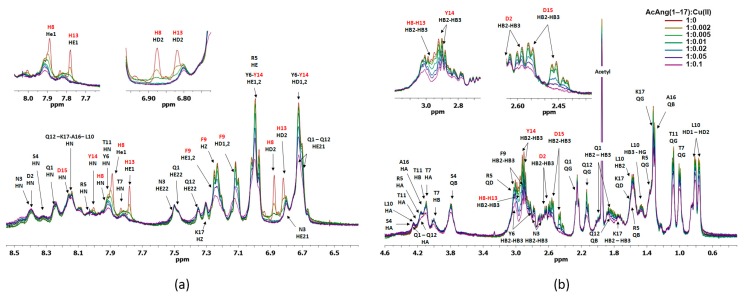
Superposition of 1H aromatic (**a**) and aliphatic (**b**) region for AcAng(1–17) peptide by increasing substechiometric metal to ligand molar ratio: 0, 1:0.002, 1:0.005, 1:0.01, 1:0.02, 1: 0.05, and 1:0.1 at pH 7. Insets: aromatic and aliphatic protons with total disappearance or broadening of the signals.

Figure 5 shows the superposition of ^1^H aromatic and aliphatic region for AcAng(1–17) by increasing sub-stoichiometric metal to ligand molar ratio, from 1:0 to 1:0.1, obtained at pH 7.

From the spectra, the gradual but specific decrease in intensity involving mainly proton signals from H8, H13 and D15 and, to a lesser extent, from F9 and Y14 residues is evident. The involvement of H8, H13 and D15 residues in metal binding is further proven by the disappearance or broadening of their protons in the ^1^H-^13^C HSQC spectra (Figure S4).

**Figure 6:**
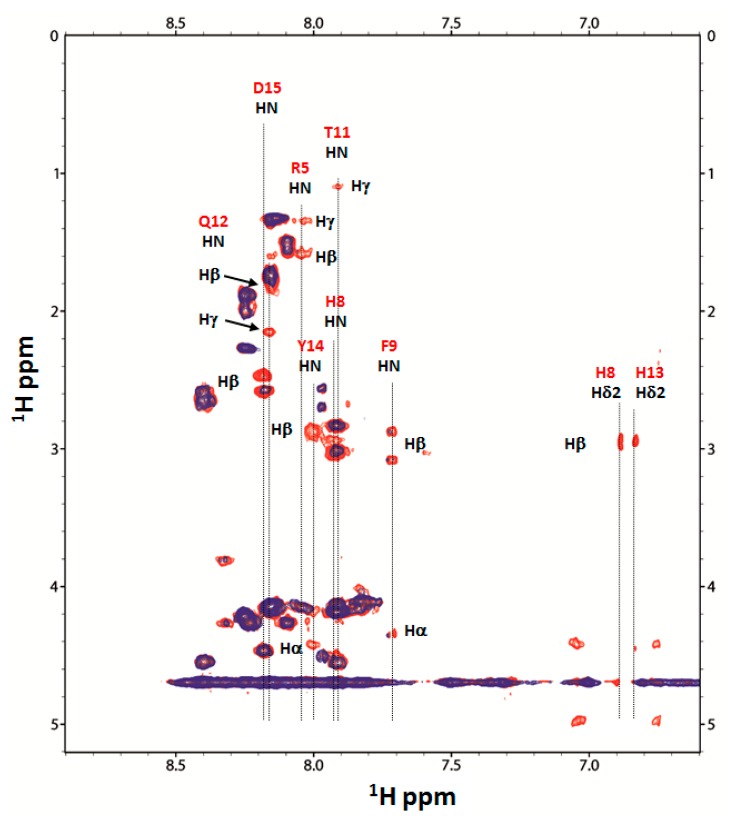
Comparison in the 1H-1H TOCSY spectrum, aromatic region, of AcAng(1–17) free (red) and AcAng(1–17):Cu(II) 1:0.05 system (blue) at pH 7.

Additional data to support the involvement of these residues in the coordination are provided by the ^1^H-^1^H TOCSY, where the spin system HN from H8, H13 and D15 in the AcAng(1–17):Cu(II) 1:0.05 system disappears (Figure 6).

A relevant line broadening is also detected for F9, T11, Q12 and Y14, indicating that the residues mainly involved in the coordination are those between H8 and H13 (Figure S5).

The NMR study of Ang(1–17) has been carried out by using the same increasing sub-stoichiometric copper to ligand molar ratios, at both pH of 5.5 and 7.

**Figure 7:**
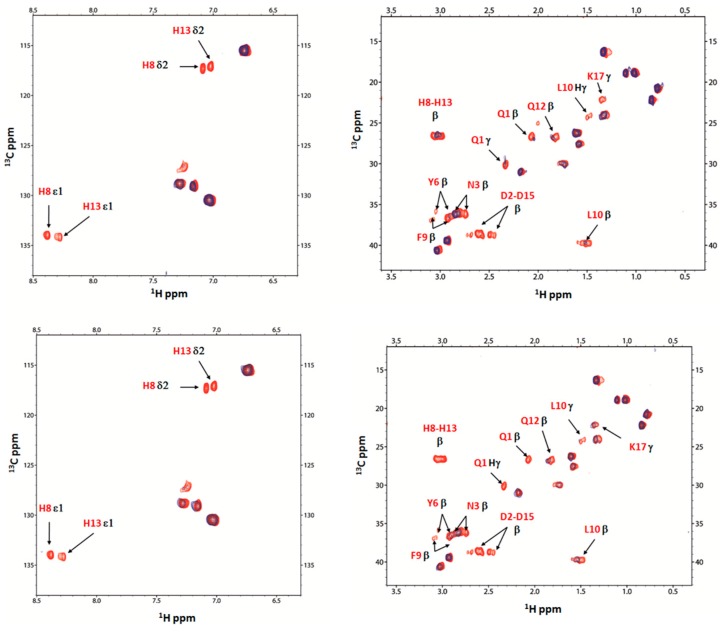
^1^H-^13^C HSQC NMR spectra, in the aromatic and aliphatic region, of Ang(1–17) free (red) and Cu(II):Ang(1–17) system at 1 to 0.02 and 1 to 0.05 (blue) molar ratio at pH 5.5.

In Figure 7, the 2D ^1^H-^13^C HSQC NMR spectra of Ang(1–17) at pH 5.5, in the aromatic and aliphatic region, for the free (in red) and the copper complex systems at 0.02/1 and 0.05/1 molar ratios (in blue) are reported. The most evident difference with respect to AcAng(1–17) is the strong decrease of Q1, D2, N3 and Y6 signals, other than Hβ of D15, Hε1 and Hδ2 of H8 and H13 residues. 

**Figure 8:**
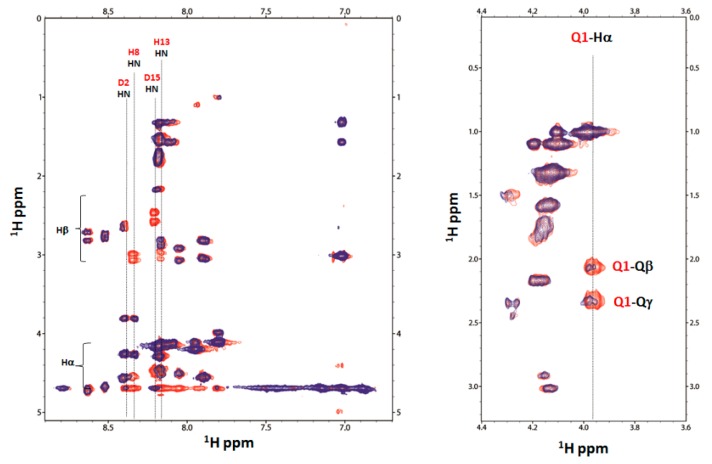
NMR ^1^H-^1^H TOCSY spectrum of Ang(1–17) free (red) and Cu(II):Ang(1–17) system at 1 to 0.02 and 1 to 0.05 (blue) molar ratio at pH 5.5.

The ^1^H-^1^H TOCSY spectra of Ang(1–17), both free and Cu(II)-complexed at pH 5.5, are reported in Figure 8. The proton spin systems of D15 residue totally disappear, whereas those of D2 residue are still present, though decreased in intensity.

The titration of Ang(1–17) with increasing amount of Cu(II), in the molar ratio range from 1:0 to 1:0.05, has been followed at pH 7 and the corresponding ^1^H spectra are reported in Figure S6. From the comparison of ^1^H-^13^C HSQC NMR spectra in the aromatic and aliphatic region of Ang(1–17) free (red) and of Cu(II):Ang system (blue) (Figure S7) and from the selection of ^1^H-^1^H TOCSY spectrum of the same systems (Figure S8), it is evident that the signals from H8 and H13 entirely disappear. Moreover, the H-C correlations from Asp residues, D2 and D15, almost disappeared in the HSQC spectrum, are instead still present in the TOCSY spectrum, suggesting that their involvement in the coordination to Cu(II) ion is reduced, mainly for D15 residue.

Line broadening can be clearly noticed for R5, F9 and Y14 residues. The residues mainly affected by the interaction of Cu(II) ion with Ang(1–17) are evidenced in Figure S9. Conversely to what has been determined with AcAng(1–17), the D15 residue in Ang(1–17) shows a limited participation in complex formation, as demonstrated by its almost unaffected resonances in the TOCSY spectra obtained at pH 7 (Figure S7). In summary, the NMR data at pH 7 indicate the involvement of both N-terminal residue (Q1, Asp2) together with imidazole, as deduced from potentiometry and UV-vis CD results.

### 2.5. Neuroblastoma Cells Experiments

**Figure 9:**
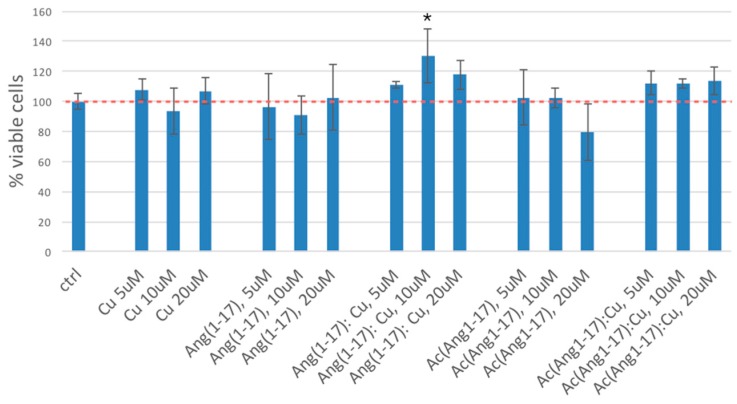
Results of MTT assay (expressed in terms of intensity normalized to the control) for SH-SY5Y cells incubated 1 h (37 °C, 5% CO_2_) with CuSO_4_, Ang(1–17), Ang(1–17):CuSO_4_ (1:1), AcAng(1–17) and AcAng(1–17):CuSO4 (1:1) in the culture medium. Data are average of three different experiments (error bar = standard deviation). Peptide concentrations: 5 × 10^−6^, 1 × 10^−5^, and 2 × 10^−5^ M. (* *p* < 0.05 of significance with respect to control, One-way Analysis of variance (ANOVA) test). The red dot line is to guide the eye at the control level.

The results of thetetrazolium dye 3-(4,5-dimethylthiazol-2-yl)-2,5-diphenyltetrazolium bromide (MTT) assay (Figure 9) evidence that all the tested treatments are non-cytotoxic. No significant differences in cell viability are found, except for the incubation with 10 µM of the complex Ang(1–17):Cu(II), where a small increase in neuroblastoma cell viability is measured compared to the control.

Preliminary cellular experiments of confocal imaging were performed to scrutinize qualitatively the effects of both peptides and the proteins in terms of actin organization, when supplemented to the cells together with the metal ions.

**Figure 10:**
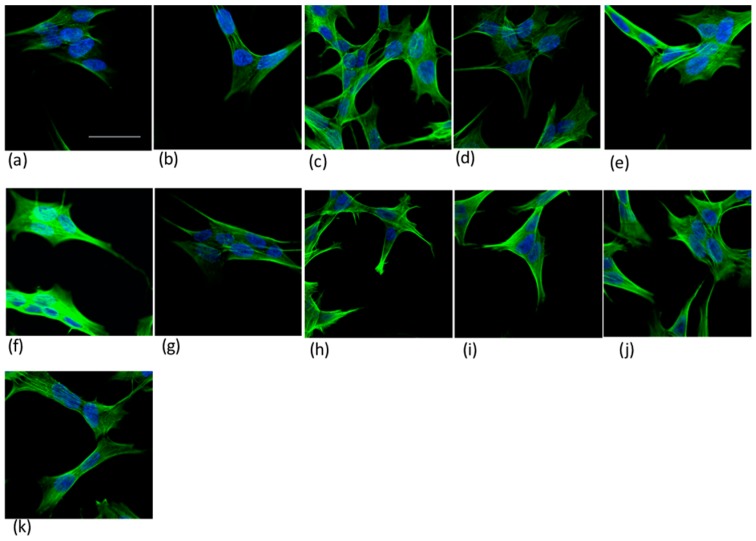
Merged confocal images recorded in the blue (DAPI staining of nuclei, λex/λem = 405/425–475 nm) and in the green (actin staining, λex/λem = 488/500–530 nm) channel for SH-SY5Y cells incubated 1 h (37 °C, 5% CO_2_) with: (**a**) 10 µM Ang(1–17); (**b**) Ang(1–17):CuSO_4_ (1:1, 10 µM); (**c**) AcAng(1–17); (**d**) AcAng(1–17):CuSO_4_ (1:1, 10 µM); (**e**) 10 µM CuSO_4_; (**f**) 100 nM r-Ang; (**g**) r-Ang:CuSO_4_ (1:1, 100 nM); (**h**) wt-Ang; (**i**) wt-Ang:CuSO_4_ (1:1, 100 nM); (**j**) 100 nM CuSO_4_; and (**k**) control. Scale bar = 30 µm.

The distribution of actin filaments in the neuroblastoma cells treated with Ang(1–17) and AcAng(1–17), both apo- and complexed-forms with Cu(II) ions, in comparison with the whole Ang protein, both wt-Ang and r-Ang, is shown in Figure 10.

The common patterns for actin microfilament organization in the cell cytoplasm, i.e., peripheral actin bands, actin bundles (stress fibers), and diffuse actin networks, are visible for all the used cell treatments. The actin pattern is brightest for the cells treated with r-Ang (Figure 10f), while it visibly changes in cells treated with r-Ang:Cu(II) complex (Figure 10g). On the contrary, both the treatments with wt-Ang (Figure 10h) and wt-Ang:Cu(II) complex (Figure 10i) induce comparable actin patterns, with an evident polygonal arrangement of actin filaments.

It is noteworthy that the cells treated with Ang(1–17):Cu(II) complex (Figure 10b) display a similar actin pattern than those treated with the free-amino r-Ang complexed to copper. On the other hand, for the acetylated peptide, similarities are found between the cells treated with the apo-form (Figure 10c) and the N terminal amino-blocked wt-Ang (Figure 10h).

**Figure 11:**
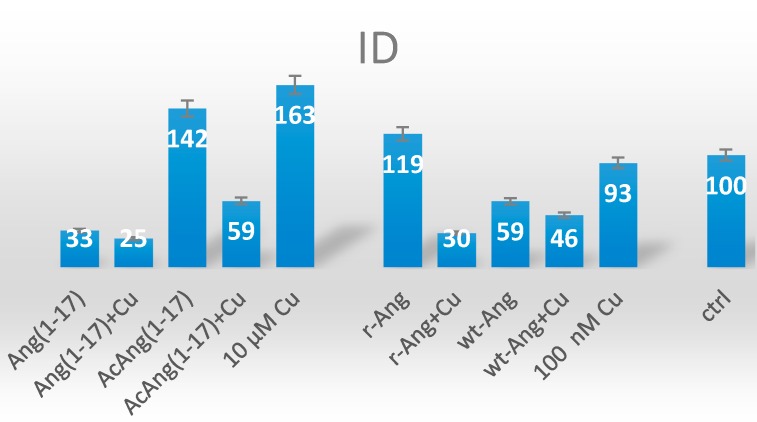
Quantitative analysis of emission recorded for actin staining (λex/λem = 488/500–530 nm) from the confocal microscopy analyses. The fluorescence intensities were calculated in terms of integrated density (ID) values. Mean ID (with the standard deviation) from five randomly chosen fields are shown. Data are normalized with respect to the control.

The quantitative analysis of fluorescence for the actin staining is shown in Figure 11.

The cells treated with the metal alone at the concentration used with the proteins (100 nM) do not significantly differ with respect to the control untreated cells. On the other hand, the cells supplemented with 10 µM copper (i.e., the concentration used for the peptides) visibly increase the actin fluorescence intensity. The overall trend observed, for both the proteins and the peptides, is a decrease of intensity for cell treatment with their copper complexes in comparison to the treatment with the free ligands. It is noteworthy that such intensity decrease exhibit similarities in wt-Ang and Ang(1–17) (i.e., huge decrease in intensity) and r-Ang and AcAng(1–17) (i.e., small decrease in intensity), which are in contrast to what was expected on the basis of the peptidomimetic design strategy, namely AcAng(1–17) mimicking wt-Ang and Ang(1–17) mimicking the r-Ang. This finding suggest that other factors are likely to be involved in the peptide-metal/cell membrane interaction which cannot simply be ruled out by this experiments. Specifically, the involvement of other protein domains and/or the formation of various Ang–Ang aggregates driven by the presence of the free amino group can be invoked. Once again, these considerations stress the relevant issue of the use of the “proper” protein form, wt-Ang, to scrutinize the pathways involving the protein in angiogenesis processes, especially in the presence of the metal ions.

## 3. Discussion

The protein angiogenin (Ang) is a potent angiogenic factor whose activity is influenced by copper ions. Many literature reports on the protein activity have been obtained using the recombinant form (r-Ang), which contains an extra methionine as first residue. In contrast, the protein effectively present in human plasma (wt-Ang), exhibit the amino terminal group blocked, owing to the spontaneously cyclization of the first residue glutamine to pyroglutamate.

To focus on the copper binding events within the N-terminal domain of the protein, we synthesized two peptides encompassing the residues 1–17 of the protein, Ang(1–17), with the amino free, and AcAng(1–17), the analogous form with the N-terminal amino group acetylated.

The chemical characterization by means of potentiometry, NMR, UV-vis and CD spectroscopies of the copper(II) complexes formed with Ang(1–17) and AcAng(1–17) demonstrate that the copper coordination environment in the N-terminal domain of the protein is strongly influenced by the free amino group. Specifically, for Ang(1–17), mimicking the recombinant protein form (r-Ang), the predominant copper complex species at physiological pH involves: (i) the amino group; (ii) the deprotonated amide of Asp-2; and (iii) one of imidazole of His-8 or His-13. On the contrary, the acetylated peptide AcAng(1–17), which has a blocked amino group in the N-terminus as the wild type protein, wt-Ang, binds copper ions through: (i) the two imidazole groups; and (ii) the deprotonated amide nitrogen atoms nearby the histidine residues. 

Taking into account the biological role of the protein in physiological as well as pathological conditions involving the angiogenic processes, we tested the activity of the two peptides in terms of cell viability and staining of actin, which is one of the potential target receptors driving the Ang–cell membrane interaction. 

Cellular experiments in neuroblastoma cell line SH-SY5Y cells demonstrate that, at the used experimental conditions, both the peptides and their correspondent copper complexes are not-cytotoxic. Rather, a small increase of cell viability, which can be explained as proliferative activity [53], is found for the cells treated with free amino peptide Ang(1–17) complexed with Cu(II) ions.

The actin patterns, followed by confocal microscopy, of the cells treated with the peptides and the proteins, in the apo- or copper-complexed form, evidence strong differences between acetylated and free-amino peptides. Indeed, notwithstanding a general trend of decreased fluorescence intensity for the copper-complexed forms with respect to the free peptide ligand, the AcAng(1–17):Cu(II) complex exhibits a huge decrease of the emission of the stained actin, whereas the effect is smoothed for Ang(1–17):Cu(II) species.

This observation is explained in terms of the different metal coordination modes and binding affinities likely determining different aggregation morphologies that might result in significantly different biological effects [54].

As to the actin staining results for the cells treated with the whole proteins, the recombinant form is more active (i.e., strong fluorescence emission) than the wild-type angiogenin. Analogously to the peptides, the respective copper complexes exhibit a decrease of fluorescence intensity, this effect being larger for r-Ang than wt-Ang.

Since one possible process related to the actin activation mechanism is the occurrence of both intramolecular Ang–Ang and intermolecular Ang–actin interactions [22], the presence of free amino groups might actually affect such a pathway. Again, the strong effect displayed by copper addition in the case of r-Ang can be correlated to the metal-chelating capability of such free amino groups, as demonstrated for short peptide sequences as well as oligopeptides [54,55]. For example, in the case of r-Ang, the formation of dimeric protein structures with bridges through the amino groups could be prompted by the presence of the copper [37].

In conclusion, this work provides experimental proof to support the significance of using the actual angiogenin present in the human plasma, the wild type form (wt-Ang), to scrutinize the protein activity, especially in the presence of the metal ions.

## 4. Materials and Methods

### 4.1. Chemicals

The peptide Ang(1–17) and Ac(ang(1–17) were supplied by Caslo Aps, Lyngby, Denmark. All other chemicals, of the highest available grade, were purchased from Sigma-Aldrich (Munich, Germany) and used without further purification.

### 4.2. Potentiometric Titrations

Potentiometric titrations were performed with a home-assembled fully automated apparatus sets (Metrohm E654 pH-meter, combined micro pH glass electrode, Orion 9103SC, Hamilton digital dispenser, Model 665) controlled by the appropriate software set up in our laboratory. The titration cell (2.5 mL) was thermostated at 298.0 ± 0.2 K, and all solutions were kept under an atmosphere of argon, which was bubbled through a solution having the same ionic strength and temperature as the measuring cell. KOH solutions (0.1 M) were added through a Hamilton buret equipped with 1 cm^3^ syringe. The ionic strength of all solutions was adjusted to 0.10 M (KNO_3_). In order to determine the stability constants, solutions of the ligands (protonation constants) or the ligands with Cu^2+^ (copper complex constants) were titrated using 0.1 M sodium hydroxide. Ligand concentration ranged from 1.4 to 2.0 × 10^−3^ for the protonation and complexation experiments, respectively. A minimum of three independent runs were performed to determine the protonation constants, while four independent experiments were run for the copper(II) complexation constants.

Metal to ligand ratios of 1:1 were employed. The initial pH was always adjusted to 2.4. To avoid systematic errors and verify reproducibility, the electromotive force (EMF) values of each experiment were taken at different time intervals.

To obtain protonation and complexation constants, the potentiometric data were refined using Hyperquad [56], which minimizes the error square sum of the measured electrode potentials through a nonlinear iterative refinement of the sum of the squared residuals, *U*, and also allows for the simultaneous refinement of data from different titrations:

*U* = Σ(*E*_exp_ − *E*_calc_)^2^

where *E*_exp_ and *E*_calc_ are the experimental and calculated electrode potentials, respectively. Errors in stability constant values are reported as three times standard deviations.

The formation reaction equilibria of ligands with protons and copper(II) ions are given in Equation (1):

pCu + qH + rL ⇆ Cu_p_H_q_L_r_ (1)

where L are the peptides under study. The stability constant β_pqr_ is defined in Equation (2):

β_pqr_ = [Cu_p_H_q_L_r_]/[Cu]_p_ · [H]_q_ · [L]_r_ (2)

The species distribution as a function of the pH was obtained using the computer program Hyss [57].

### 4.3. Ultraviolet-Visible (UV-vis) Measurements

UV-vis spectra were recorded at 25 °C using an Agilent 8453 or a Varian Cary 500 spectrophotometer (Agilent Technologies, Santa Clara, CA, USA). The concentrations of the peptides and copper(II) used to record absorption spectra were the same as those for the potentiometric titrations. Combined spectroscopic and potentiometric metal-complex titrations were performed into a 3 mL quartz cuvette with a 1 cm path length to obtain the spectrum in the Visible region at each pH value simultaneously. These experiments were replicated at least three times for each copper-peptide system. Spectroscopic data were processed by means of HYPERQUAD program [56].

### 4.4. Circular Dichroism (CD) Measurements

CD spectra were obtained at 25 °C under a constant flow of nitrogen on a Jasco model 810 spectropolarimeter (Jasco, Easton, MD, USA) at a scan rate of 50 nm·min^−1^ and a resolution of 0.1 nm, the path length being 1 cm, in the 280–800 nm range. The spectra were recorded as an average of either 3 or 5 scans. Calibration of the instrument was performed with a 0.06% aqueous solution of ammonium camphorsulfonate. The CD spectra of the copper(II) complexes on varying the solution pH were obtained in both the 190–250 and 250–800 nm wavelength regions. All the solutions were freshly prepared using double distilled water. The copper(II) ion and peptide concentrations used for the acquisition of the CD spectra in the Visible region were identical to those used in the potentiometric titrations. The results are reported as ε (molar adsorption coefficient) and Δε (molar dichroic coefficient) in M^−1^·cm^−1^.

### 4.5. Nuclear Magnetic Resonance (NMR) Spectroscopy

NMR experiments were carried out on a Bruker Ascend^TM^ 400 MHz spectrometer (Bruker, Billerica, MA, USA) equipped with a 5 mm automated tuning and matching broad band probe (BBFO) with z-gradients, as previously described [58,59].

NMR measurements were performed by using a concentration of the peptides of 2 mM, in 90/10 (*v*/*v*) H_2_O/D_2_O, at 298 K. HSQC (2D ^1^H-^13^C heteronuclear correlation spectra) were acquired using a phase-sensitive sequence utilizing Echo-Antiecho-TPPI gradient selection with a heteronuclear coupling constant JXH = 145 Hz, and shaped pulses for all 180° pulses on f2 channel with decoupling during acquisition. Sensitivity improvement and gradients in back-inept were also used. In all of the experiments, relaxation delays of 2 s and 90° pulses of about 10 µs were used. The solvent suppression in the 1D ^1^H, 2D ^1^H-^1^H TOCSY and 2D ^1^H-^1^H ROESY experiments was performed by using excitation sculpting with gradients. The spin-lock mixing time of TOCSY experiments was obtained with MLEV17. ^1^H-^1^H TOCSY spectra were carried out using 60 ms as mixing times. The signals of both free and metal-bound peptides at different pH values and different metal to ligand molar ratios have been assigned by using a combination of 1D, 2D TOCSY, HSQC and ROESY experiments. All NMR results were processed by using TopSpin (Bruker Instruments) software and analyzed using Sparky 3.11 and MestRe Nova 6.0.2 (Mestrelab Research S.L.) programs (Santiago de Compostela, Spain).

### 4.6. Cellular Experiments

Human neuroblastoma SH-SY5Y cells were grown in DMEM-F12 (1:1) medium, supplemented with 10% fetal bovine serum, 1% penicillin/streptomycin, and 2 mM l-glutamine, and maintained in a humidified incubator at 37 °C in 5% CO_2_ atmosphere.

#### 4.6.1. Cell Viability Assay

In order to evaluate the proliferative and pro-survival effects of Ang(1–17) and AcAng(1–17) peptides, the colorimetric MTT assay was performed. The assay is based on the reduction, worked by cellular oxidoreductase enzymes that reflect the number of viable cells present, of the tetrazolium dye MTT 3-(4,5-dimethylthiazol-2-yl)-2,5-diphenyltetrazolium bromide to its insoluble formazan, which has a purple color.

Cells were plated in a 48-well plate and grown in DMEM-F12 up to 70%–80% confluency, followed by treatment for 24 h with each of the two peptides at the concentration of 5 × 10^−6^, 1 × 10^−5^ and 2 × 10^−5^ M, as well as the correspondent complexes with CuSO_4_ at equimolar concentrations. MTT (1 mg/mL) in PBS was then added to wells and incubated at 37 °C for 2 h to allow for complete cleavage of the tetrazolium salt by metabolically active cells. Next, MTT was removed and 220 µL of dimethyl sulfoxide (DMSO) was added. One hundred microliters of each well was transferred to a 96-well plate followed by colorimetric analysis using a multilabel plate reader at 560 nm of wavelength. Absorbance values plotted are the mean from triplicate experiments. Statistical relevance was calculated by One-way Analysis of variance (ANOVA) test performed with Origin 8.3 software (Northampton, MA, USA).

#### 4.6.2. Confocal Microscopy Imaging

SH-SY5Y cells in culture at 80% of confluence were split on glass bottom Petri dishes (WillCo Wells, glass diameter of 22 mm). The day after cells were treated for 1 h with Ang(1–17), AcAng(1–17), wt-Ang, r-Ang, CuSO_4_, the peptide–copper and the protein–copper complexes at 1:1 mole ratio. The used concentrations were of 1 × 10^−7^ M for the peptides and 1 × 10^−5^ M for the proteins.

After the incubation time, cells were washed with phosphate buffer saline solution (10 mM PBS, 37 °C, pH = 7.4), fixed with high purity 4% formaldehyde in PBS (pH = 7.3) and stained with the nuclear dye DAPI (ThermoFisher). Afterwards, cells were permeabilized with 0.5% Triton X-100 and stained with a high-affinity F-actin probe, conjugated to green-fluorescent Alexa Fluor^®^ 488 dye (ActinGreen™ 488 ReadyProbes^®^ Reagent, TermoFisher (Thermo Fisher Scientific, Waltham, MA, USA).

Confocal imaging was performed with an Olympus FV1000 confocal laser scanning microscope (LSM, Olympus, Shinjuku, Japan), equipped with diode UV (405 nm, 50 mW), multiline Argon (457, 488, 515 nm, total 30 mW), HeNe(G) (543 nm, 1 mW) and HeNe(R) (633 nm, 1 mW) lasers. An oil immersion objective (60xO PLAPO) and spectral filtering system were used. The detector gain was fixed at a constant value and images were taken, in sequential mode, for all the samples at random locations throughout the area of the well. Quantitative analysis of fluorescence was performed using the ImageJ software (1.50i version, NIH), in terms of integrated density ID = *N*·[*M* − *B*], where *N* is the number of pixels in the selection, *M* is the average gray value of the pixels and *B* is the most common pixel value [60].

## Figures and Tables

**Table 1:** Protonation constant (logβ_qr_) and p*K* values of (Ang(1–17) and AcAng(1–17) (*T* = 298 K and *I* = 0.1 M KNO_3_). ^a^

Species_qr_	logβ_qr_^b^	logβ_qr_^b^
	L = Ang(1–17)	L = AcAng(1–17)
HL	7.18 (2)	10.89 (1)
H_2_L	13.60 (2)	20.90 (1)
H_3_L	19.45 (2)	29.87 (3)
H_4_L	23.28 (3)	36.39 (5)
H_5_L	26.55 (2)	42.34 (4)
H_6_L	-	46.24 (5)
H_7_L	-	49.63 (4)
p*K* COO^−^	3.27	3.39
p*K* COO^−^	3.83	3.90
p*K* His	5.84	5.95
p*K* His	6.42	6.52
p*K* NH_2_	7.18	-
p*K* Tyr or Lys	-	8.97
p*K* Tyr or Lys	-	10.01
p*K* Tyr or Lys	-	10.89

^a^ Standard deviations (3σ values) are given in parentheses; [L] = 1 × 10^−3^ M; ^b^ qH + rL = H_q_L_r_; β_qr_ = [H_q_L_r_]/[H]^q^[L]^r^.

**Table 2:** Stability constants (logβ_pqr_) and p*K* values of copper(II) complexes with Ang(1–17) and AcAng(1–17); (T = 298 K, I = 0.1 M KNO_3_). ^a^

Species (pqr)	logβ_pqr_^b^ L = Cu–Ang(1–17)	p*K*(n/m)	Species (pqr)	logβ_pqr_^b^ L = Cu–AcAng(1–17)	p*K*(n/m)
CuLH	13.23 (1)	-	CuLH_4_	40.38 (3)	-
CuL	6.80 (2)	(1/0) = 6.43	CuLH_3_	35.82 (1)	(4/3) = 4.56
CuLH_−1_	−1.34 (2)	(0/−1) = 8.14	CuLH_2_	29.52 (3)	(3/2) = 6.30
CuLH_−2_	−10.39 (2)	(−1/−2) = 9.04	CuLH	23.73 (1)	(2/1) = 5.79
CuLH_−3_	−19.85 (2)	(−2/−3) = 9.46	CuL	16.35 (2)	(1/0) = 7.38
CuLH_−4_	−30.12 (2)	(−3/−4) = 10.25	CuLH_−1_	7.66 (2)	(−1/0) = 8.69
CuLH_−5_	−40.04 (2)	(−4/−5) = 9.94	CuLH_−2_	−2.16 (2)	(−2/−1) = 9.82
			CuLH_−3_	−12.36 (2)	(−3/−2) = 10.20

^a^ Standard deviations (3σ values) are given in parentheses. Charges are omitted for clarity; p*K*(n/m) values reflect the p*K* value of copper(II) complexes; [L] = 1 × 10^−3^ M; molar ratio 1:1; ^b^ pCu + qH + rL = Cu_p_H_q_L_r_; β_bqr_ = [Cu_p_H_q_L_r_]/[Cu]^p^[H]^q^[L]^r^.

**Table 3:** Spectroscopic parameters of Copper(II) complexes.

L	pH	Species (CuLH)	UV-vis λ (nm) (ε, M^−1^·cm^−1^) ^a^	CD λ (nm) (Δε, M^−1^·cm^−1^)
Ang(1–17)	5.5	CuLH	628 (90)	265 (1.171); 298 (−1.224); 620 (−0.223)
	7	CuL	605 (95)	264 (3.28); 299 (−1.318); 336 (0.456); 597 (−0.456)
	8.5	CuLH_−1_	574 (106)	265 (3.847); 297 (0.305) 322 (1.051); 574 (−0.704)
	9	CuLH_−1_ CuLH_−2_ CuLH_-3_	-	263 (3.805); 315 (1.409); 563 (−0.875)
	10	CuLH_−3_ CuLH_−3_ CuLH_−4_	-	263 (3.225); 309 (1.675); 363 (−0.221); 555 (−1.023)
	11	CuLH_−5_	525 (130)	262 (3.051); 308 (1.454); 352 (−0.314); 555 (−1.056)
AcAng(1–17)	5	Cu (40%), CuLH_4_ (20%), CuLH_3_ (40%)	-	619 (0.163)
	5.9	CuLH_3_	650 (60)	258 (2.518); 331 (0.304); 598 (−0.236)
	6.5	CuLH_3_; CuLH_2_; CuLH	-	256 (7.449); 330 (0.602); 526 (0.203); 594 (−0.403)
	7.5	CuLH	585 (105)	262 (8.525); 323 (1.031); 365 (−0.127); 502 (−0.745); 678 (0.621)
	8.3	CuL	540 (125)	263 (7.999); 318 (1.143); 359 (-0.524); 500 (−1.127); 648 (0.989)
	9.5	CuLH_-1_;	540 (135)	264 (7.812); 310 (1.360); 352 (−0.791); 501 (−1.216); 641 (1.276)
	10.2	CuLH_-2_	540 (128)	264 (7.622); 312 (1.252); 351 (−0.789); 502 (−1.192); 648 (1.262)
	11	CuLH_−2_; CuLH_−3_	540 (135)	264 (7.115); 312 (1.181); 350 (−0.825); 501 (−1.161); 641 (1.269)

^a^ Ref. [42].

## Supplementary Materials

Supplementary materials can be found at http://www.mdpi.com/1422-0067/17/8/1240/s1.
