# Supplementary material for: Coordination Environment of Cu(II) Ions Bound to N-Terminal Peptide Fragments of Angiogenin Protein
Source: Int J Mol Sci. 2016 Aug 1;17(8):1240. doi: 10.3390/ijms17081240 (PMC5000638; doi:10.3390/ijms17081240)
Supplement: Supplementary file 1 [file ijms-17-01240-s001.pdf]

Supplementary Material: Coordination Environment of Cu(II) Ions Bound to N-Terminal Peptide Fragments of Angiogenin Protein

Antonio Magrì, Alessia Munzone, Massimiliano Peana, Serenella Medici, Maria Antonietta Zoroddu, Orjan Hansson, Cristina Satriano, Enrico Rizzarelli and Diego La Mendola

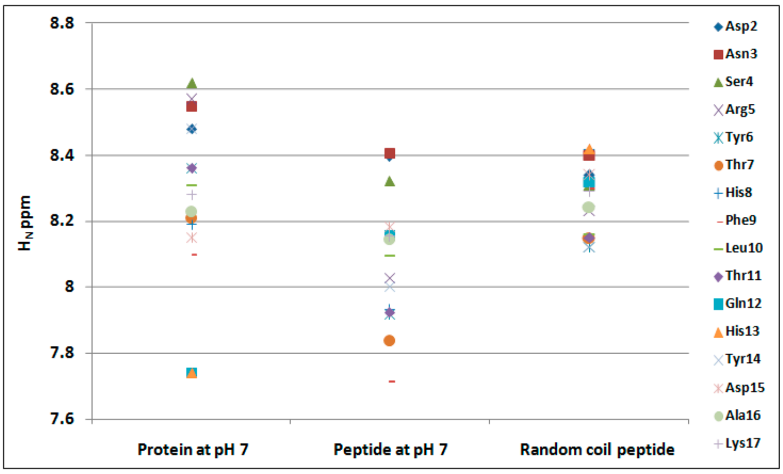

Figure S1. Comparison of the spread HN chemical shift (ppm) values for the fragment 1–17 in the protein, in the peptides 1–17 and in a random coil conformation.

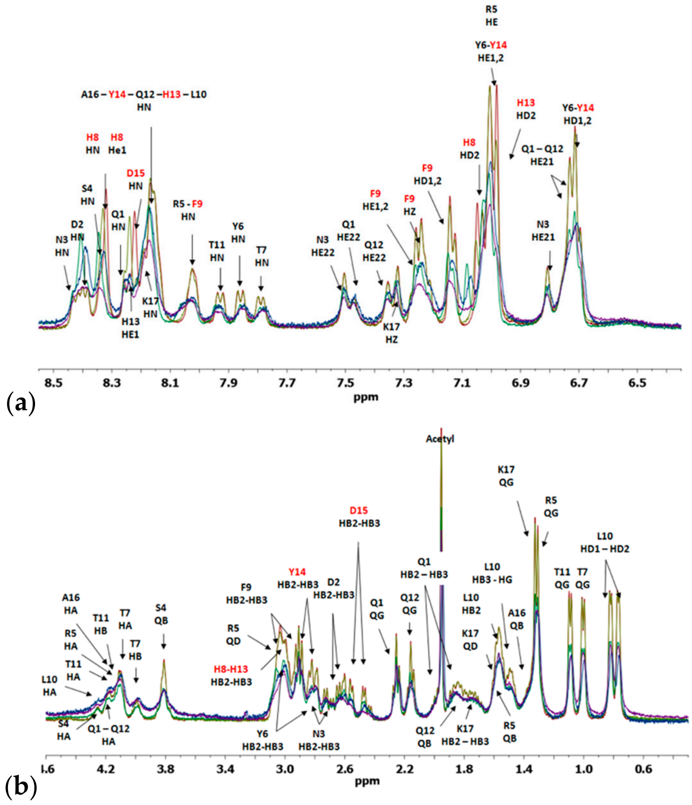

Figure S2. (**a**,**b**). Superposition of 1H aromatic and aliphatic region for AcAng(1–17) peptide by increasing metal to ligand molar ratio: 1:0, 1:0.002, 1:0.005, 1:0.01, 1:0.02 at pH 5.5. Signals more affected by copper titration are reported in red.

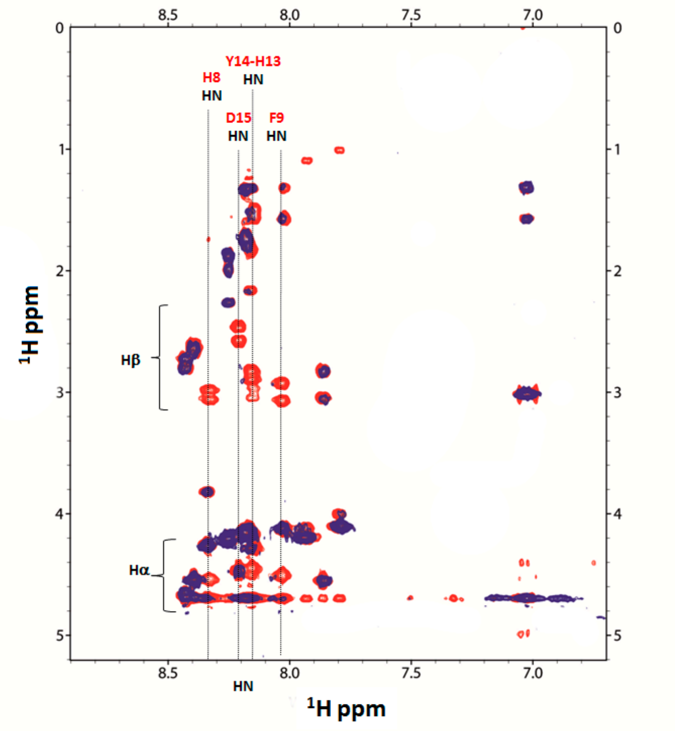

Figure S3. Comparison in the 1H-1H TOCSY spectra, aromatic region, for AcAng(1–17) (red) and AcAng(1–17):Cu(II) (blue) system 1:0.02 at pH 5.5. The disappearing spin system HN for H8, F9, H13, Y14 and D15 have been labelled.

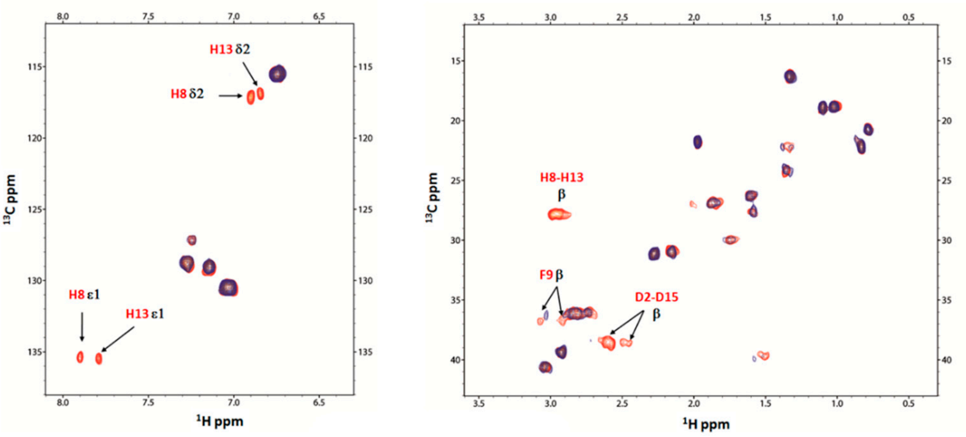

Figure S4. Aromatic and aliphatic region of 1H-13C HSQC of AcAng(1–17) free (red) and AcAng(1–17):Cu(II) 1:0.05 (blue) at pH 7. Disappearing peaks and those with major broadening have been labelled.

Ac-$Q_1D_2N_3S_4R_5Y_6T_7H_8F_9L_{10}T_{11}Q_{12}H_{13}Y_{14}D_{15}A_{16}K_{17}$

Ac-$Q_1D_2N_3S_4R_5Y_6T_7H_8F_9L_{10}T_{11}Q_{12}H_{13}Y_{14}D_{15}A_{16}K_{17}$

Figure S5. Residues mainly affected in AcAng(1–17) sequence by Cu(II) coordination at pH 5.5 and 7 are highlighted in the sequence. Residues showing: major broadening (red); broadening (blue-light).

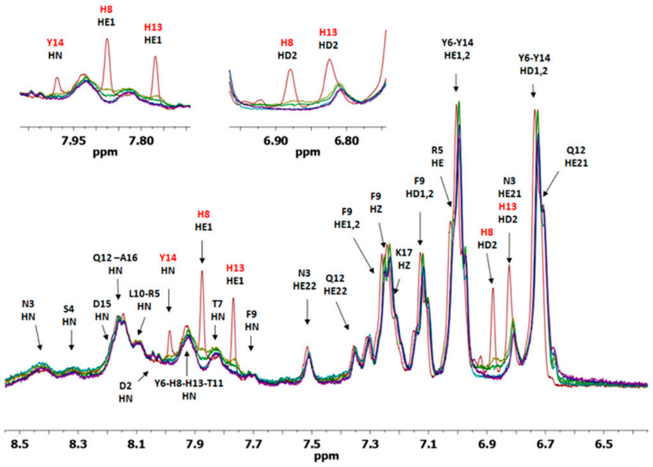

(**a**)

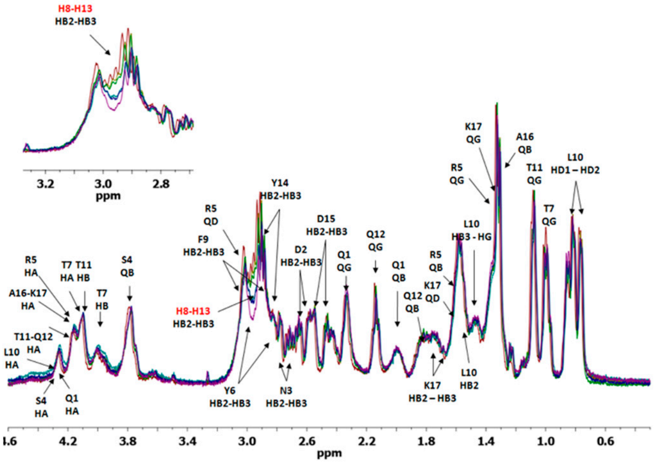

(**b**)

Figure S6. (**a**,**b**) Superposition of 1H aromatic and aliphatic region for Ang(1–17) peptide by increasing Cu(II) to ligand molar ratio: 1:0, 1:0.002, 1:0.005, 1:0.01, 1:0.02, 1: 0.05 at pH 7. Signals more affected by copper titration are reported in red.

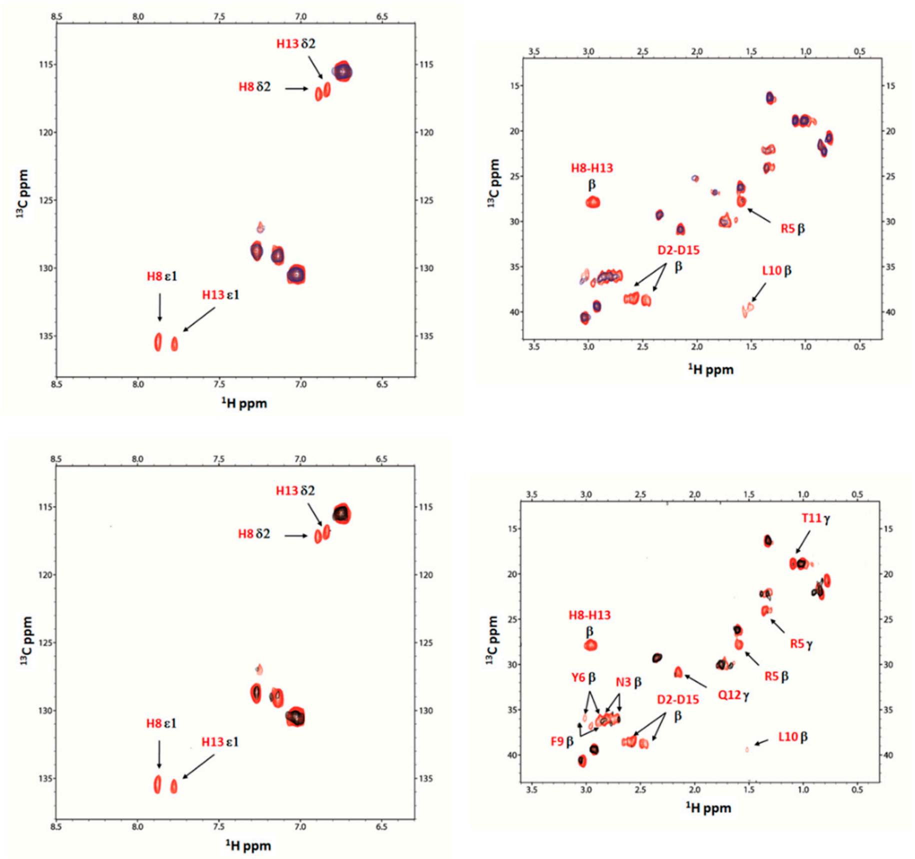

Figure S7. 1H-13C HSQC NMR spectra in the aromatic and aliphatic region, of Ang(1–17) free (red) with Cu(II): Ang(1–17) system at 1 to 0.02 and 1 to 0.05 (blue) molar ratio at pH 7.

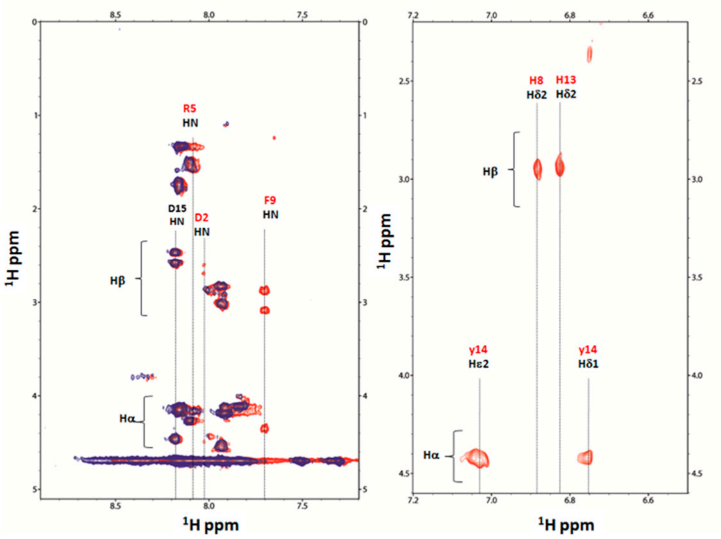

Figure S8. Comparison in the 1H-1H TOCSY spectra, aromatic region, for Ang(1–17) free (red) and Ang(1–17):Cu(II) (blu) system 1:0.05 at pH 7. The disappearing signals for H8, F9, H13, Y14 have been labelled in red. HN system of D15 is visible and labelled in black.

NH_2-$Q_1D_2N_3S_4R_5Y_6T_7H_8F_9L_{10}T_{11}Q_{12}H_{13}Y_{14}D_{15}A_{16}K_{17}$

NH_2-$Q_1D_2N_3S_4R_5Y_6T_7H_8F_9L_{10}T_{11}Q_{12}H_{13}Y_{14}D_{15}A_{16}K_{17}$

Figure S9. Residues mainly affected in Ang sequence by Cu(II) coordination at pH 5.5 and 7 are highlighted in the sequence. Residues showing: major broadening (red); broadening (blue-light); and partial broadening (orange).

Table S1. ^{1}H proton resonances assignment for Ac-Ang peptide at pH 5.5.

Group	Atom	Shift	Group	Atom	Shift	Group	Atom	Shift	Group	Atom	Shift
Q1	HN	8252	R5	QD	3016	F9	HE1	7278	H13	HE1	8237
Q1	HA	4212	Y6	HN	7863	F9	HE2	7270	Y14	HN	8160
Q1	HB2	1997	Y6	HA	4548	F9	HZ	7238	Y14	HA	4447
Q1	HB3	1879	Y6	HB2	3041	L10	HN	8148	Y14	HB2	2885
Q1	QG	2261	Y6	HB3	2830	L10	HA	4280	Y14	HB3	2827
Q1	HE21	6761	Y6	HD1	6736	L10	HB2	1576	Y14	HD1	6731
Q1	HE22	7471	Y6	HD2	6730	L10	HB3	1495	Y14	HD2	6736
D2	HN	8392	Y6	HE1	7031	L10	HG	1486	Y14	HE1	7029
D2	HA	4540	Y6	HE2	7026	L10	HD1	832	Y14	HE2	7029
D2	HB2	2660	T7	HN	7795	L10	HD2	776	D15	HN	8210
D2	HB3	2627	T7	HA	4105	T11	HN	7932	D15	HA	4468
N3	HN	8424	T7	HB	4007	T11	HA	4199	D15	HB2	2573
N3	HA	4672	T7	QG	1012	T11	HB	4115	D15	HB3	2481
N3	HB2	2824	H8	HN	8331	T11	QG	1096	A16	HN	8168
N3	HB3	2732	H8	HA	4535	Q12	HN	8159	A16	HA	4134
N3	HE21	6812	H8	HB2	3055	Q12	HA	4186	A16	QB	1325
N3	HE22	7508	H8	HB3	2992	Q12	HB3	1840	K17	HN	8181
S4	HN	8339	H8	HD2	7056	Q12	QG	2167	K17	HA	4144
S4	HA	4260	H8	HE1	8334	Q12	HE21	6742	K17	HB2	1765
S4	QB	3821	F9	HN	8032	Q12	HE22	7358	K17	HB3	1707
R5	HN	8024	F9	HA	4509	H13	HN	8152	K17	HZ	7325
R5	HE	7029	F9	HB2	3066	H13	HA	4524	K17	QG	1346
R5	HA	4129	F9	HB3	2933	H13	HB2	3052	K17	QD	1593
R5	QB	1579	F9	HD1	7143	H13	HB3	2996	K17	QE	2914
R5	QG	1319	F9	HD2	7161	H13	HD2	6992			

Table S2. ^{13}C carbon resonances assignment for Ac-Ang peptide at pH 5.5.

Group	Atom	Shift	Group	Atom	Shift	Group	Atom	Shift
Q1	CA	53.296	T7	CG	18.867	Q12	CB	26.845
Q1	CB	26.845	H8	CB	26.531	Q12	CG	30.915
Q1	CG	31.089	H8	CD2	117.329	H13	CB	26.738
D2	CA	52.397	H8	CE1	134.090	H13	CD2	117.096
D2	CB	38.387	F9	CA	55.156	H13	CE1	134.245
N3	CB	36.019	F9	CB	36.576	Y14	CA	55.060
S4	CA	56.914	F9	CD1	129.059	Y14	CB	36.230
S4	CB	60.840	F9	CD2	129.064	Y14	CD1	115.501
R5	CA	54.093	F9	CE1	128.792	Y14	CD2	115.501
R5	CB	27.535	F9	CE2	128.788	Y14	CE1	130.493
R5	CG	24.138	F9	CZ	127.294	Y14	CE2	130.493
R5	CD	40.642	L10	CA	52.684	D15	CA	50.802
Y6	CA	55.475	L10	CB	39.776	D15	CB	38.686
Y6	CB	35.998	L10	CD1	22.189	A16	CA	50.192
Y6	CD1	115.501	L10	CD2	20.749	A16	CB	16.351
Y6	CD2	115.501	L10	CG	24.111	K17	CA	53.661
Y6	CE1	130.493	T11	CA	59.278	K17	CB	30.061
Y6	CE2	130.493	T11	CB	67.026	K17	CG	22.183
T7	CA	59.502	T11	CG	18.892	K17	CD	26.271
T7	CB	67.007	Q12	CA	53.340	K17	CE	39.395

Table S3. [1]H proton resonances assignment for Ac-Ang peptide at pH 7.

Group	Atom	Shift	Group	Atom	Shift	Group	Atom	Shift	Group	Atom	Shift
Q1	HN	8248	Y6	HN	7916	F9	HZ	7236	Y14	HB3	2846
Q1	HA	4215	Y6	HA	4553	L10	HN	8095	Y14	HD1	6744
Q1	HB2	2000	Y6	HB2	3025	L10	HA	4261	Y14	HD2	6744
Q1	HB3	1883	Y6	HB3	2836	L10	HB2	1556	Y14	HE1	7029
Q1	QG	2267	Y6	HD1	6744	L10	HB3	1496	Y14	HE2	7029
Q1	HE21	6761	Y6	HD2	6738	L10	HG	1483	D15	HN	8182
Q1	HE22	7473	Y6	HE1	7029	L10	HD1	828	D15	HA	4470
D2	HN	8398	Y6	HE2	7020	L10	HD2	777	D15	HB2	2572
D2	HA	4541	T7	HN	7836	T11	HN	7923	D15	HB3	2479
D2	HB2	2657	T7	HA	4109	T11	HA	4189	A16	HN	8143
D2	HB3	2612	T7	HB	4016	T11	HB	4117	A16	HA	4132
N3	HN	8405	T7	QG	1015	T11	QG	1096	A16	QB	1329
N3	HA	4682	H8	HN	7934	Q12	HN	8158	K17	HN	8155
N3	HB2	2803	H8	HA	4504	Q12	HA	4187	K17	HA	4147
N3	HB3	2729	H8	HB2	2962	Q12	QB	1847	K17	HB2	1760
N3	HE21	6812	H8	HB3	2944	Q12	QG	2153	K17	HB3	1744
N3	HE22	7507	H8	HD2	6890	Q12	HE21	6742	K17	QG	1342
S4	HN	8322	H8	HE1	7897	Q12	HE22	7357	K17	QD	1592
S4	HA	4268	F9	HN	7712	H13	HA	4443	K17	QE	2915
S4	QB	3814	F9	HA	4353	H13	HB2	2953	K17	HZ	7310
R5	HN	8026	F9	HB2	3074	H13	HB3	2947			
R5	HA	4155	F9	HB3	2896	H13	HD2	6834			
R5	QB	1591	F9	HD1	7141	H13	HE1	7787			
R5	QG	1348	F9	HD2	7141	Y14	HN	8002			
R5	QD	3023	F9	HE1	7271	Y14	HA	4431			
R5	HE	7030	F9	HE2	7269	Y14	HB2	2882			

Table S4. [13]C carbon resonances assignment for Ac-Ang peptide at pH 7.

Group	Atom	Shift	Group	Atom	Shift	Group	Atom	Shift
Q1	CA	53,120	H8	CB	27,806	H13	CD2	116,943
Q1	CB	26,928	H8	CD2	117,101	H13	CE1	135,459
Q1	CG	31,153	H8	CE1	135,375	Y14	CA	55,186
D2	CA	51,826	F9	CA	52,276	Y14	CB	36,265
D2	CB	38,430	F9	CB	36,730	Y14	CD1	115,514
N3	CB	36,081	F9	CD1	129,042	Y14	CD2	115,514
S4	CA	56,857	F9	CD2	129,042	Y14	CE1	130,514
S4	CB	60,796	F9	CE1	128,773	Y14	CE2	130,514
R5	CA	53,983	F9	CE2	128,773	D15	CA	51,238
R5	CB	27,579	F9	CZ	127,178	D15	CB	38,621
R5	CD	40,578	L10	CA	52,989	A16	CA	50,298
R5	CG	24,222	L10	CB	39,675	A16	CB	16,287
Y6	CA	55,117	L10	CD1	22,264	K17	CA	53,661
Y6	CB	36,115	L10	CD2	20,750	K17	CB	29,967
Y6	CD1	115,514	T11	CA	59,453	K17	CD	26,321
Y6	CD2	115,514	T11	CB	66,952	K17	CE	39,420
Y6	CE1	130,514	T11	CG	18,908	K17	CG	22,268
Y6	CE2	130,514	Q12	CA	53,340			
T7	CA	59,616	Q12	CB	26,863			
T7	CB	66,907	Q12	CG	30,946			
T7	CG	18,859	H13	CB	27,806			

Table S5. ^{1}H proton resonances assignment for NH_2-Ang peptide at pH 5.5.

Group	Atom	Shift	Group	Atom	Shift	Group	Atom	Shift	Group	Atom	Shift
Q1	HA	3966	Y6	HN	7900	L10	HN	8156	Y14	HD1	6734
Q1	QB	2070	Y6	HA	4551	L10	HA	4282	Y14	HD2	6734
Q1	QG	2333	Y6	HB2	3039	L10	HB2	1561	Y14	HE1	7027
D2	HN	8397	Y6	HB3	2826	L10	HB3	1504	Y14	HE2	7027
D2	HA	4562	Y6	HD1	6734	L10	HG	1484	D15	HN	8211
D2	HB2	2664	Y6	HD2	6726	L10	HD1	830	D15	HA	4473
D2	HB3	2611	Y6	HE1	7027	L10	HD2	781	D15	HB2	2573
N3	HN	8523	Y6	HE2	7024	T11	HN	7938	D15	HB3	2481
N3	HA	4691	T7	HN	7808	T11	HA	4199	A16	HN	8168
N3	HB2	2820	T7	HA	4115	T11	HB	4112	A16	HA	4141
N3	HB3	2730	T7	HB	3991	T11	QG	1096	A16	QB	1324
N3	HE21	6815	T7	QG	1008	Q12	HN	8174	K17	HN	8182
N3	HE22	7514	H8	HN	8346	Q12	HA	4179	K17	HA	4150
N3'	HN	8629	H8	HA	4543	Q12	QB	1836	K17	HB2	1761
N3'	HA	4698	H8	HB2	3053	Q12	QG	2167	K17	HB3	1706
N3'	HB2	2817	H8	HB3	2987	Q12	HE21	6746	K17	QG	1352
N3'	HB3	2720	H8	HD2	7075	Q12	HE22	7359	K17	QD	1593
S4	HN	8337	H8	HE1	8375	H13	HN	8170	K17	QE	2917
S4	HA	4264	F9	HN	8053	H13	HA	4528	K17	HZ	7325
S4	QB	3811	F9	HA	4513	H13	HB2	3050	K17	HX?	7006
S4	HN'	8394	F9	HB2	3066	H13	HB3	2984			
R5	HN	8075	F9	HB3	2932	H13	HD2	7008			
R5	HA	4132	F9	HD1	7144	H13	HE1	8271			
R5	QB	1579	F9	HD2	7156	Y14	HN	8172			
R5	QD	3017	F9	HE1	7277	Y14	HA	4454			
R5	QG	1322	F9	HE2	7268	Y14	HB2	2899			
R5	HE	7022	F9	HZ	7235	Y14	HB3	2827			

Table S6. ^{13}C carbon resonances assignment for NH_2-Ang peptide at pH 5.5.

Group	Atom	Shift	Group	Atom	Shift	Group	Atom	Shift
Q1	CA	52,504	T7	CG	18,894	Q12	CB	26,845
Q1	CB	26,714	H8	CB	26,531	Q12	CG	30,915
Q1	CG	30,202	H8	CD2	117,291	H13	CB	26,738
D2	CA	52,622	H8	CE1	133,994	H13	CD2	117,099
D2	CB	38,710	F9	CA	55,156	H13	CE1	134,162
N3	CB	36,158	F9	CB	36,699	Y14	CA	55,270
S4	CA	56,856	F9	CD1	129,080	Y14	CB	36,280
S4	CB	60,805	F9	CD2	129,080	Y14	CD1	115,480
R5	CA	54,111	F9	CE1	128,793	Y14	CD2	115,480
R5	CB	27,535	F9	CE2	128,793	Y14	CE1	130,474
R5	CG	23,945	F9	CZ	127,233	Y14	CE2	130,474
R5	CD	40,608	L10	CA	52,646	D15	CA	50,920
Y6	CA	55,142	L10	CB	39,774	D15	CB	38,686
Y6	CB	35,850	L10	CG	24,111	A16	CA	50,174
Y6	CD1	115,480	L10	CD1	22,217	A16	CB	16,339
Y6	CD2	115,480	L10	CD2	20,748	K17	CA	53,749
Y6	CE1	130,474	T11	CA	59,245	K17	CB	29,963
Y6	CE2	130,474	T11	CB	67,062	K17	CG	22,239
T7	CA	59,466	T11	CG	18,936	K17	CD	26,271
T7	CB	66,986	Q12	CA	53,340	K17	CE	39,361

Table S7. [1]H proton resonances assignment for NH_2-Ang peptide at pH 7.

Group	Atom	Shift	Group	Atom	Shift	Group	Atom	Shift	Group	Atom	Shift
Q1	HA	4282	Y6	HN	7949	F9	HD1	7140	H13	HN	7928
Q1	QB	1995	Y6	HA	4569	F9	HD2	7137	H13	HA	4441
Q1	QG	2341	Y6	HB2	3013	F9	HE1	7272	H13	HB2	2947
D2	HN	8029	Y6	HB3	2829	F9	HE2	7266	H13	HB3	2944
D2	HA	4540	Y6	HD1	6744	F9	HZ	7248	H13	HD2	6829
D2	HB2	2668	Y6	HD2	6737	L10	HN	8098	H13	HE1	7776
D2	HB3	2604	Y6	HE1	7026	L10	HA	4279	Y14	HN	7993
N3	HN	8462	Y6	HE2	7021	L10	HB2	1569	Y14	HA	4429
N3	HA	4693	T7	HN	7828	L10	HB3	1510	Y14	HB2	2886
N3	HB2	2799	T7	HA	4120	L10	HG	1480	Y14	HB3	2849
N3	HB3	2711	T7	HB	4006	L10	HD1	828	Y14	HD1	6751
N3	HE21	6816	T7	QG	1011	L10	HD2	780	Y14	HD2	6745
N3	HE22	7515	H8	HN	7928	T11	HN	7922	Y14	HE1	7025
S4	HN	8305	H8	HA	4505	T11	HA	4188	Y14	HE2	7026
S4	HA	4273	H8	HB2	2954	T11	HB	4116	D15	HN	8177
S4	QB	3799	H8	HB3	2953	T11	QG	1096	D15	HA	4470
R5	HN	8075	H8	HD2	6885	Q12	HN	8156	D15	HB2	2572
R5	HA	4167	H8	HE1	7882	Q12	HA	4179	D15	HB3	2481
R5	QB	1591	F9	HN	7699	Q12	QB	1837	A16	HN	8137
R5	QG	1363	F9	HA	4371	Q12	QG	2149	A16	HA	4132
R5	QD	3029	F9	HB2	3071	Q12	HE21	6740	A16	QB	1331
R5	HE	7030	F9	HB3	2880	Q12	HE22	7357			

Table S8. [13]C carbon resonances assignment for NH_2-Ang peptide at pH 7.

Group	Atom	Shift	Group	Atom	Shift	Group	Atom	Shift
Q1	CA	54,810	H8	CA	53,172	Q12	CG	30,757
Q1	CB	25,271	H8	CB	27,814	H13	CA	55,268
Q1	CG	29,270	H8	CD2	117,192	H13	CB	27,794
D2	CA	51,488	H8	CE1	135,497	H13	CD2	116,871
D2	CB	38,560	F9	CA	55,140	H13	CE1	135,602
N3	CB	35,983	F9	CB	36,177	Y14	CA	55,036
S4	CA	56,802	F9	CD1	129,059	Y14	CB	36,177
S4	CB	60,929	F9	CD2	129,025	Y14	CD1	115,530
R5	CA	53,922	F9	CE1	128,737	Y14	CD2	115,530
R5	CB	27,816	F9	CE2	128,755	Y14	CE1	130,466
R5	CG	24,025	F9	CZ	126,999	Y14	CE2	130,466
R5	CD	40,647	L10	CA	52,689	D15	CA	51,241
Y6	CA	55,415	L10	CB	39,430	D15	CB	38,549
Y6	CB	35,904	L10	CG	24,129	A16	CA	50,369
Y6	CD1	115,528	L10	CD1	22,290	A16	CB	16,367
Y6	CD2	115,530	L10	CD2	20,724	K17	CA	53,749
Y6	CE1	130,469	T11	CA	59,448	K17	CB	30,084
Y6	CE2	130,466	T11	CB	66,982	K17	CG	22,095
T7	CA	59,570	T11	CG	18,874	K17	CD	26,197
T7	CB	66,911	Q12	CA	53,340	K17	CE	39,402
T7	CG	18,837	Q12	CB	26,871			
